# Effectiveness and safety of vascular intervention plus lenvatinib versus vascular intervention alone for hepatocellular carcinoma patients with portal vein tumor thrombus: a retrospective comparative study

**DOI:** 10.3389/fonc.2024.1431069

**Published:** 2024-07-05

**Authors:** Saikang Tang, Yingming Gao, Xue Yan, Weihua Zhi, Yue Han

**Affiliations:** ^1^ Department of Interventional Therapy, National Cancer Center/National Clinical Research Center for Cancer/Cancer Hospital, Chinese Academy of Medical Sciences and Peking Union Medical College, Beijing, China; ^2^ Department of General Surgery, Cancer Hospital of Huanxing, Beijing, China

**Keywords:** hepatocellular carcinoma (HCC), portal vein tumor thrombus (PVTT), transarterial chemoembolization (TACE), hepatic arterial infusion chemotherapy (HAIC), lenvatinib

## Abstract

**Background:**

This study aimed to assess the effectiveness and safety of vascular intervention combined with lenvatinib versus vascular intervention alone in the treatment of advanced hepatocellular carcinoma (HCC) with portal vein tumor thrombus (PVTT), and to identify prognostic factors associated with the treatment outcomes.

**Methods:**

We conducted a retrospective analysis of data from 92 patients with advanced HCC and PVTT who were treated between February 2016 and February 2023. Among them, 56 patients underwent vascular intervention alone (transarterial chemoembolization, TACE), while 36 patients received vascular intervention (TACE or hepatic arterial infusion chemotherapy [HAIC]) combined with lenvatinib. The primary outcomes included progression-free survival (PFS), overall survival (OS), and objective response rate (ORR). Survival rates were estimated by the Kaplan-Meier method, and confounders were adjusted using inverse probability of treatment weighting (IPTW). Prognostic factors were determined through the Cox regression model.

**Results:**

The median follow-up duration was 20.07 months (interquartile range: 6.41–25.36). The combination therapy group had a significantly longer median PFS (11.00 vs. 5.00 months, P<0.05) and OS (12.91 vs. 6.83 months, P<0.05) in comparison to the monotherapy group, and these findings remained consistent after IPTW matching. Moreover, the combination therapy group showed a higher ORR (55.56% vs. 26.79%, P<0.05) based on mRECIST criteria. Cox multivariate analysis identified extrahepatic metastasis and maximum tumor diameter as risk factors for PFS, while age, tumor number, and maximum tumor diameter influenced OS. Combined treatment emerged as a protective factor for OS. In the combination therapy group, hypertension was the most frequent adverse event, with grade 3 or 4 adverse events occurring rarely.

**Conclusion:**

The combination of vascular intervention with lenvatinib has demonstrated improved PFS and OS in advanced HCC patients with PVTT, and its safety profile appears to be acceptable. Adoption of this combined treatment strategy at an earlier stage may enhance patient outcomes.

## Introduction

Hepatocellular carcinoma (HCC) is the sixth most prevalent cancer worldwide, and has the third highest mortality rate, surpassed only by lung and colorectal cancer (1, 2). In China, it ranks fourth in cancer incidence and second in terms of mortality (3, 4), with a five-year survival rate of only 12.1% (5). Early-stage HCC goes undetected due to the absence of clinical symptoms, resulting in a diagnosis at an advanced stage in 70%-80% of patients. Among them, 44%-62.2% of patients present with portal vein tumor thrombus (PVTT), which is associated with an exceedingly unfavorable prognosis, with a median survival of only 2.7 months (6, 7).

PVTT results in extensive dissemination of tumors throughout the liver, causing portal hypertension, hepatocellular jaundice, and refractory ascites. When the main portal vein is affected, it significantly disrupts the portal venous blood supply, leading to severe liver function deterioration, rapid disease progression, and ultimately liver function decompensation. This, in turn, deprives patients of the opportunity for curative treatment (8–10).

Vascular interventional therapies, such as transarterial chemoembolization (TACE) and hepatic arterial infusion chemotherapy (HAIC), alongside systemic therapies like lenvatinib and sorafenib, are frequently employed in clinical practice for the management of patients with unresectable HCC and concurrent PVTT (11–13).

TACE has emerged as the standard therapeutic approach for intermediate and advanced-stage HCC. In China, HAIC is primarily recommended for patients who either decline or demonstrate insufficient response to systemic therapy, as well as those with selective extrahepatic metastases. Japanese scholars endorse HAIC for patients who have experienced failure or resistance to TACE (14). However, TACE treatment might induce hypoxia, increasing the expression of vascular endothelial growth factor (VEGF), which might be an important factor contributing to TACE resistance. And other factors also contribute to the complex tumor microenvironment of HCC (15, 16).

Lenvatinib, a multi-kinase inhibitor, exerts inhibitory effects on vascular endothelial growth factor receptor (VEGFR) and platelet-derived growth factor receptor (PDGFR), thereby suppressing VEGF and PDGF signaling. This mechanism promotes tumor shrinkage, necrosis, and reduces portal vein pressure (17, 18). This mechanism might also reduce TACE resistance, suggesting a potential synergistic effect between lenvatinib and TACE treatment (19). Lenvatinib has demonstrated efficacy as a treatment modality for reducing tumor burden and regressing PVTT (20).

The available clinical research evidence strongly supports the utilization of combination therapy as a comprehensive treatment approach for advanced-stage liver cancer, surpassing monotherapy with either vascular intervention or lenvatinib. The combination therapy group has shown superior objective response rate (ORR), progression-free survival (PFS), and overall survival (OS), while maintaining an acceptable safety profile (19–22).

This study was conducted to evaluate the effectiveness and safety of combined therapy involving lenvatinib and vascular intervention compared to vascular intervention alone in patients with advanced hepatocellular carcinoma (HCC) and concurrent portal vein tumor thrombus (PVTT), and to identify prognostic factors associated with treatment outcomes.

## Materials and methods

### Patient

Data were retrospectively collected from advanced HCC patients with PVTT who underwent treatment at the Department of Interventional Therapy, Cancer Hospital, Chinese Academy of Medical Sciences, between February 2016 and February 2023. A total of 92 patients with complete data were included in this study. Among them, 56 patients underwent vascular interventional treatment (monotherapy group), while 36 patients underwent vascular intervention combined with lenvatinib (combination therapy group).

The study applied the following inclusion criteria: (1) patients diagnosed with HCC through clinical evaluation and further confirmed with imaging evidence of PVTT; (2) patients undergoing the combination therapy of vascular interventional treatment (TACE or HAIC) with lenvatinib or TACE alone; (3) presence of at least one lesion that can be assessed using Response Evaluation Criteria in Solid Tumors (RECIST) version 1.1 and modified RECIST (mRECIST); (4) Child-Pugh class A or B; (5) Eastern Cooperative Oncology Group performance status (ECOG-PS) 0 and 1. The criteria for the combination period were established to administer lenvatinib concurrently with TACE/HAIC therapy or within a 60-day window before or after TACE/HAIC treatment.

The exclusion criteria were as follows: (1) presence of liver metastases originating from malignancies in other organs or intrahepatic cholangiocarcinoma; (2) lesions that could not be measured; (3) absence of baseline or follow-up imaging data; (4) coexistence of other malignancies.

Clinical data for each patient were collected, including age, gender, body mass index (BMI), hepatitis B/hepatitis C infection status, Child-Pugh class, ECOG score, and baseline alpha-fetoprotein (AFP) level. Tumor-related variables were also recorded, such as the number of primary tumors, maximum diameter, PVTT classification [Cheng’s classification method (23, 24)], and presence of extrahepatic metastasis.

This study was approved by the Ethics Committee of the Cancer Hospital, Chinese Academy of Medical Sciences, ensuring adherence to the ethical standards established by the institutional and/or national research committee (Ethics Approval Number:23/518–4261). The procedures were conducted in accordance with the principles outlined in the 1964 Helsinki Declaration and its subsequent amendments. The Ethics Committee granted a waiver for individual informed consent.

### Clinical treatment procedure

All patients underwent vascular interventional therapy. The monotherapy group received TACE, while the combination therapy group received TACE/HAIC along with lenvatinib. TACE involved the identification of tumor-feeding arteries through angiography, followed by the intrarterial administration of chemotherapy drugs and iodized oil. The chemotherapy regimen included agents such as paclitaxel, oxaliplatin, lobaplatin, and hydroxycamptothecin, with embolization performed using iodized oil and/or gelatin sponge particles. The HAIC treatment protocol involved the direct placement of a catheter into the hepatic artery, either directly or immediately following TACE treatment. Continuous 24-hour infusion chemotherapy of oxaliplatin, calcium folinate, and fluorouracil was administered through a subcutaneously implanted port system.

In clinical practice, the selection of treatment modality is based on the evaluation of the patient’s condition and comprehensive evaluation of disease. TACE was primarily indicated for patients exhibiting minor PVTT and a limited tumor burden. Conversely, HAIC was contemplated for cases involving advanced tumor thrombus classified as type III or IV, large tumor burden, and scenarios where TACE was deemed insufficient for achieving optimal embolization due to the presence of significant arteriovenous shunts. Additionally, HAIC was considered for patients presenting with concurrent distant metastasis.

The dosage of lenvatinib was determined based on the patient’s body weight, with a daily dose of 12 mg for individuals weighing ≥60 kg and 8 mg for those weighing <60 kg. Lenvatinib administration was discontinued on the day of each interventional treatment. If the interventional treatment did not result in significant symptoms such as fever, nausea, or vomiting, lenvatinib was resumed after the intervention. In cases where the intervention caused significant and persistent symptoms, lenvatinib treatment was resumed after symptom relief. The drug label allowed for lenvatinib dosage reduction (to 8 mg and 4 mg per day) to mitigate drug-related toxicity.

### Adverse events

AEs occurring during lenvatinib treatment were primarily assessed by their frequency and severity, following the Common Terminology Criteria for Adverse Events (CTCAE, version 5.0). Mild and transient AEs, such as pain, fever, elevated liver enzymes, and nausea, occurring after vascular interventional therapy were not documented for the patients.

### Follow-up and outcome assessment

Follow-up visits were scheduled at approximately 6-week intervals, involving enhanced CT or MRI scans. OS denoted the duration between the initial vascular intervention or lenvatinib treatment after PVTT diagnosis and either death or last follow-up. PFS represented the timeframe from the initial lenvatinib administration or vascular intervention and disease progression or last follow-up. PFS and OS rates for 6 and 12 months were calculated. RECIST 1.1 and mRECIST criteria were employed to assess effectiveness, yielding the objective response rate (ORR) and disease control rate (DCR). Tumor reactions were categorized as complete response (CR), partial response (PR), stable disease (SD), or progressive disease (PD). ORR encompassed the combined rates of CR and PR, while DCR combined ORR with SD rate.

### Statistical analysis

Qualitative data were analyzed using the chi-square or Fisher’s exact test, while quantitative data were subjected to the t-test or Wilcoxon rank-sum test. Kaplan-Meier analysis was employed to evaluate OS and PFS rates. To mitigate potential selection bias between the two groups, inverse probability of treatment weighting (IPTW) was used. Prognostic factors in advanced HCC patients with PVTT were evaluated through Cox regression model. A significance level of P < 0.05 was deemed statistically significant. Data analysis was conducted using R 4.2.2 for Windows.

## Results

### Baseline characteristics

Ninety-two patients were enrolled in the study, with a median age of 59 years (interquartile range [IQR]: 48.75–64.25). Most were male, accounting for 92.39% (n = 85). The median BMI was 23.6 kg/m^2^ (IQR: 21.175–26.225), and there was a significant difference between the two groups (24.6 vs. 22.35, P < 0.05). Hepatitis B or C virus infection was detected in 95.6% (n = 87) of the patients with 80.34% (n = 74) classified as Child-Pugh class A. The baseline AFP levels were 419.75 ng/ml (IQR: 29.14–18061.50). The majority of patients (65.93%) had three or more liver tumor lesions, with a maximum tumor diameter of 8.7 cm (IQR: 6.00–12.25). Lymph node or distant metastasis was observed in 40.22% (n = 37) of the patients. Fifteen patients (16.30%) had received prior treatments before intervention/lenvatinib therapy, and there was a significant difference between the 2 groups (8.93% vs. 27.78%). **Table 1** shows the detailed patient characteristics.

**Table 1:** Baseline demographics and clinical characteristics in 92 patients.

Characteristics	Total (N=92)	Monotherapy Group (N_1_ = 56)	Combination Therapy Group (N_2_ = 36)	P
Age, years, median (IQR)	59(48.75,64.25)	58(46.75,63.25)	60(55,65.25)	0.0536
BMI, median (IQR)	23.6(21.175,26.225)	24.6(22.575,26.65)	22.35(20.7,24.3)	0.0086
Gender, n (%)				
Male	85(92.39)	52(92.86)	33(91.67)	1.0000
Female	7(7.61)	4(7.14)	3(8.33)	
HBV/HCV infection, n (%)				
Presence	87(95.6)	54(98.18)	33(91.67)	0.2966
HBV	83(95.4)	53(98.15)	30(90.91)	
HCV	4(4.6)	1(1.85)	3(9.09)	
Absence	4(4.4)	1(1.82)	3(8.33)	
EHS, n (%)				
LNM	20(21.74)	12(21.43)	8(22.22)	0.9719
DM	17(18.48)	10(17.86)	7(19.44)	
Absence	55(59.78)	34(60.71)	21(58.33)	
Other treatment before vascular intervention or Lenvatinib, n (%)				
Presence	15(16.3)	5(8.93)	10(27.78)	0.0358
Absence	77(83.7)	51(91.07)	26(72.22)	
Tumor number, n (%)				
1	22(24.18)	14(25)	8(22.86)	0.5210
2	9(9.89)	7(12.5)	2(5.71)	
>=3	60(65.93)	35(62.5)	25(71.43)	
Max tumor size, cm, median (IQR)	8.7(6.00,12.25)	9.45(6.175,12.250)	7.9(5.45,11.75)	0.1941
Child-Pugh Grade, n (%)				
A	74(80.43)	42(75)	32(88.89)	0.1708
B	18(19.57)	14(25)	4(11.11)	
C	0(0)	0(0)	0(0)	
PVTT type, n (%)				
I	15(16.48)	7(12.5)	8(22.86)	0.4408
II	51(56.04)	35(62.5)	16(45.71)	
III	20(21.98)	11(19.64)	9(25.71)	
IV	5(5.49)	3(5.36)	2(5.71)	
ECOG PS score, n (%)				
0	67(72.83)	42(75)	25(69.44)	0.5588
1	25(27.17)	14(25)	11(30.56)	
Baseline AFP, ng/ml, median (IQR)	419.75(29.14,18061.50)	1648.5(53.63,18061.50)	115.3(14.49,13709.25)	0.2412

AFP, alpha-fetoprotein; BMI, body mass index; DM, distant metastases; ECOG, Eastern Cooperative Oncology Group; EHS, extrahepatic spread; HBV, hepatic B virus; HCV, hepatic C virus; IQR, interquartile range; LNM, lymph node metastases; PS, performance status.

### Effectiveness assessment

**Figure 1:**
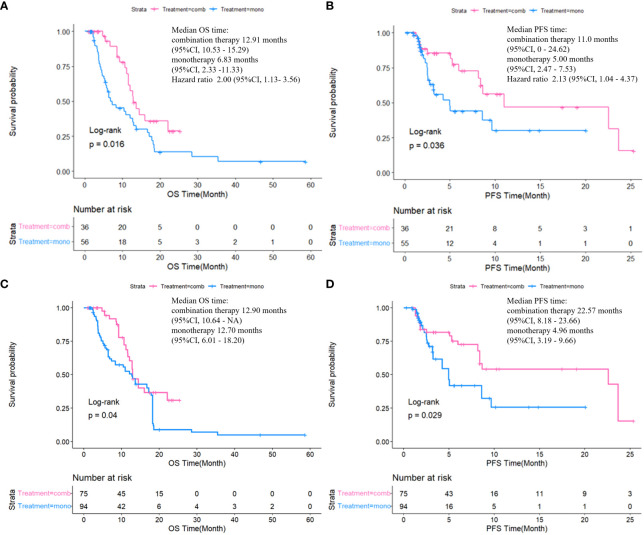
Comparison of OS and PFS between two groups by Kaplan-Meier method before **(A, B)** and after **(C, D)** IPTW.

The median follow-up duration was 20.07 months (interquartile range [IQR]: 6.41–25.36), with no notable differences in follow-up duration between the two groups (P=0.156). After the exclusion of four samples with missing values, the analysis incorporated a total of 88 samples. The combination therapy group exhibited significantly longer median PFS (mPFS) compared to the monotherapy group (11.00 vs. 5.00 months, P < 0.05), with a similar trend observed for median OS (mOS) (12.91 vs. 6.83 months, P < 0.05). The combination therapy group exhibited notably higher 6-month OS rates compared to the monotherapy group (94.44% vs. 66.07%, P<0.05) (**Supplementary Table 1**). Additionally, baseline age, BMI, and prior treatment before intervention/lenvatinib therapy were included as variables in the IPTW analysis. After IPTW analysis, the mPFS of the combination therapy surpassed that of the monotherapy group (22.57 vs. 4.96 months, P < 0.05), with the similar positive outcomes observed in mOS (12.90 vs. 12.70 months, P < 0.05). The survival curves before and after IPTW are illustrated in **Figure 1**.

In combination group, there was no significant survival difference between patients treated with TACE and those treated with HAIC (mPFS: 8.64 vs. 23.66 months, P = 0.61; mOS: 12.1 vs. 16.0 months, P =0.62) (**Supplementary Figure 1**).

In univariate Cox regression analyses, extrahepatic metastasis (lymph nodes) and maximum tumor diameter were identified as risk factors for PFS, while combined treatment demonstrated a protective effect on PFS (**Supplementary Table 2**). Tumor number (≥3), and maximum tumor diameter emerged as risk factors for OS, while combined treatment acted as a protective factor for OS (**Supplementary Table 3**). Multivariate Cox regression analysis revealed that extrahepatic metastasis (including lymph nodes and distant metastasis) and maximum tumor diameter were risk factors for PFS. However, prior treatment before intervention/lenvatinib therapy was found to be a protective factor for PFS. Furthermore, risk factors for OS encompassed age, tumor number, and maximum tumor diameter, with combined treatment exhibiting a protective effect on OS.

**Figure 2:**
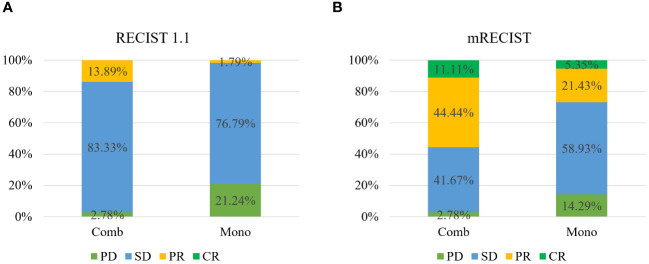
Tumor response to therapy according to RECIST v1.1 **(A)** and mRECIST **(B)** between the two groups.

The ORR based on mRECIST criteria in the combination therapy group reached 55.56%, significantly higher than the data in the monotherapy group (26.79%, P < 0.05). Besides, the ORR based on RECIST 1.1 criteria in the combination therapy group reached 13.89%, and the DCR stood at 97.22%, both markedly surpassing those in another group (ORR: 1.79%, DCR: 78.58%, P < 0.05) (**Table 2**, **Figure 2**).

**Table 2:** Tumor response to therapy according to RECIST v1.1 and mRECIST between the two groups.

	RECIST1.1			mRECIST		
	Comb(n=36)	Mono(n=56)	P	Comb(n=36)	Mono(n=56)	P
CR	0(0%)	0(0%)	–	4(11.11%)	3(5.35%)	0.426
PR	5(13.89%)	1(1.79%)	<0.05	16(44.44%)	12(21.43%)	<0.05
SD	30(83.33%)	43(76.79%)	0.449	15(41.67%)	33(58.93%)	0.106
PD	1(2.78%)	12(21.42%)	<0.05	1(2.78%)	8(14.29%)	0.084
ORR(CR+PR)	5(13.89%)	1(1.79%)	<0.05	20(55.56%)	15(26.79%)	<0.05
DCR(CR+PR+SD)	35(97.22%)	44(78.58%)	<0.05	35(97.22%)	48(85.71%)	0.084

Come, Combination therapy group; CR, Complete Response; DCR, Disease Control Rate; Mono, Monotherapy group; ORR, Overall Response Rate; PD, Progressive Disease; PR, Partial Response; RECIST, Response Evaluation Criteria In Solid Tumors; mRECIST, modified Response Evaluation Criteria In Solid Tumors; S, Stable Disease.

### Safety

As the majority of patients experienced mild and transient reactions, such as pain, fever, elevated liver enzymes, and nausea following vascular intervention therapy, which generally resolved with symptomatic treatment or short-term clinical observation, AEs subsequent to the intervention were not systematically documented.

**Table 3** presents a summary of AEs associated with oral administration of lenvatinib in patients. The most commonly reported AEs were hypertension (36.11%) and fatigue (30.56%). Apart from two cases of grade 3 hypertension, the remaining AEs were classified as grade 1 or 2.

**Table 3:** Adverse events in 36 patients receiving lenvatinib treatment.

Adverse event	Any grade	Grade 3-4
Hypertension	13(36.11)	1(2.78)
Fatigue	11(30.56)	0(0)
Decreased appetite	9(25.00)	0(0)
Diarrhea	7(19.44)	0(0)
Nausea	5(13.89)	0(0)
Abdominal pain	4(11.11)	0(0)
Hand-foot skin reaction	4(11.11)	0(0)
Dysarthria	3(8.33)	0(0)
Fever	2(5.56)	0(0)
Constipation	1(2.78)	0(0)

Values are presented as n (%).

## Discussion

Advanced-stage HCC patients with PVTT constitute a cohort characterized by severe portal hypertension, diminished liver function, and rapid disease progression. These factors contribute to a median survival of only 2.7 months, greatly impacting overall patient prognosis (6, 7, 25). Such patients are typically unsuitable for immediate curative surgery or liver transplantation, and immediate survival benefits may not be realized from these interventions (8, 10, 12, 26). The BCLC guidelines (11) recommend systemic therapy, while vascular interventional therapies such as TACE and HAIC are endorsed in Asian countries (12, 13, 26–28). However, vascular intervention alone offers limited survival extension, and its efficacy can be influenced by PVTT type and liver function grade (28, 29).

Lenvatinib has demonstrated a median survival of 13.6 months in patients with unresectable HCC (30), which has garnered recommendations from both the BCLC (11) and Chinese guidelines (6, 7) for HCC with PVTT. Regarding combination therapy involving lenvatinib plus vascular intervention, substantial progress has been made. The LAUNCH study (19) highlighted the superiority of this combination over lenvatinib alone in advanced HCC, showcasing improved mOS and mPFS. Echoing these findings, a retrospective study (22) reported superior survival outcomes with combination therapy compared to TACE alone. Our study focuses on HCC patients with PVTT, aiming to discern the differences in effectiveness between monotherapy and combination therapy, and to identify the target patient population for optimized treatment outcomes.

Our results indicate a pronounced survival benefit with the combination therapy, showcasing superior mPFS and mOS compared to vascular intervention alone. Even with the implementation of the IPTW method to mitigate selection bias, our results remained robust, underscoring the reliability of our findings. The synergy between the two treatment modalities, particularly anti-VEGF action of lenvatinib, plays a pivotal role in these outcomes (27, 31–34). Additionally, combination therapy appears to preserve liver function and reduce the frequency of required TACE sessions (22, 35). In our study, the combination therapy group achieved a mPFS of 11.0 months, which was comparable to the findings from other similar large-sample studies (10.6 months, 8.6 months) (19, 21). However, the mOS of 12.91 months was shorter than observed in other studies (17.8 months, 15.9 months) (19, 21). This discrepancy may be attributed to the heightened risk of distant metastasis in our PVTT patient cohort (36, 37), with 41.66% exhibiting extrahepatic metastasis.

The 6-month OS rate in combination group was markedly higher than that in the monotherapy group, and the 12-month OS rate, as well as the 6-month and 12-month PFS rates, also showed numerical improvement in the former. This highlights the potential importance of combination therapy in clinical practice. Furthermore, our 12-month OS rate in combination therapy group was lower compared to that in an aforementioned study (72.22% vs. 88.4%), and the 12-month PFS rate was similarly shorter than reported in that study (66.67% vs. 78.4%) (22). This can also be explained by the impact of PVTT on prognosis. Study by Yuan et al. (38), which also focused on PVTT, showed a similar 1-year OS rate (75.4%) to ours.

The classification of PVTT types revealed that the majority of patients had milder vascular invasion (Types I and II), aligning with prior research (23, 39). Our multivariate analysis indicated that patients with types III or IV PVTT did not show worse impact on OS and PFS, compared to those with type I or II. This finding contrasts with clinical observations that suggest more severe PVTT classifications correlate with poorer prognosis. In clinical practice, intervention treatments like TACE or HAIC are only recommended for patients with type III or IV PVTT if their liver function is relatively good and can tolerate such treatments. These patients generally have better conditions and more opportunities for treatment, implying greater chances of survival. This suggests that carefully selected patients with type III or IV PVTT could also potentially benefit from combined interventional and lenvatinib treatment. However, further research is necessary to validate these results, and the effects of other accompanying treatments and confounding factors should be analyzed. Yuan’s study also found that patients with Vp1–2 and Vp3–4 PVTT undergoing combined treatment (TACE-HAIC and tyrosine kinase inhibitor [TKI] plus immunotherapy) did not show a significant difference in prognosis. The researchers speculated that the prognostic impact of the severity of PVTT might be counteracted by the significant efficacy of the combined treatment (38).

In terms of treatment response, combination therapy demonstrated a superior ORR and DCR, suggesting its extensive applicability for HCC patients with PVTT. Compared with Yuan’s research, our study had an equivalent level of ORR (55.56% vs. 53.7%, based on mRECIST).

The Cox multivariate regression analysis identified extrahepatic metastasis and tumor size as risk factors for PFS, while age, tumor number, and tumor size emerged as risk factors for OS. The LAUNCH study (19), employing univariate and multivariate analyses, indicated that tumor number, microvascular invasion (MVI), and AFP level are risk factors for PFS. This aligns with our study, both elucidating that unfavorable characteristics of the tumor closely correlate with worse prognosis. Real-world data from China showed that, compared to starting lenvatinib after two vascular interventions, initiating lenvatinib before or after the first vascular intervention significantly improved mPFS in patients (14.5 vs. 8.9 months, P=0.048) (40). All these findings underscore the potential benefits of early initiation of combination therapy.

Moreover, the Cox multivariate regression analysis showed that combination therapy is a protective factor for OS. Similarly, both the LAUNCH (19) and CHANCE001 (41) studies have indicated that the combined treatment is a protective factor for OS and PFS. These findings imply that the combined therapy, as opposed to monotherapy with either targeted drugs or vascular intervention, is associated with enhanced survival benefits, bearing significant clinical implications.

Concerning safety, lenvatinib was generally well-tolerated, and hypertension was the most common AE. Grage 3 or 4 AEs were rare and manageable.

Despite these insights, our study is not without limitations. The inherent selection bias in retrospective studies raises concerns, although we employed the IPTW method to mitigate this issue. Another problem is that not many patients received HAIC treatment alone, so only patients receiving TACE treatment were included in the monotherapy group. To test the impact of different interventional techniques on patient survival within the combination therapy group, additional analyses about survival of the two groups of patients was conducted, and the results showed no significant differences. Additionally, the single-center design and limited sample size necessitate further validation through multicenter, prospective, large-sample cohort studies.

It is worth noting that immune checkpoint inhibitors have also become a standard option in the treatment of advanced HCC. The exploration of combining TACE with immunotherapy and TKIs is underway, with preliminary results indicating a synergistic effect (41, 42). The combination could potentially represent a novel treatment strategy for HCC in the future. We will also continue to explore the clinical practice of immunotherapy in combination treatments in the future.

In conclusion, our study suggests that combination therapy involving vascular intervention and lenvatinib offers a viable, relatively safe treatment option for advanced HCC patients with PVTT. It holds the potential to improve both PFS and OS compared to vascular intervention alone. Early consideration of this combined therapeutic approach may enhance patient outcomes.

## Data availability statement

The original contributions presented in the study are included in the article/**Supplementary Material**. Further inquiries can be directed to the corresponding author.

## Ethics statement

The studies involving humans were approved by Ethics Committee of the Cancer Hospital, Chinese Academy of Medical Sciences. The studies were conducted in accordance with the local legislation and institutional requirements. The participants provided their written informed consent to participate in this study.

## Author contributions

ST: Writing – original draft, Writing – review & editing, Conceptualization, Data curation, Formal analysis, Investigation, Methodology. YG: Data curation, Formal analysis, Investigation, Writing – original draft. XY: Writing – original draft, Data curation, Formal analysis, Investigation. WZ: Writing – review & editing, Data curation, Formal analysis. YH: Formal analysis, Project administration, Supervision, Writing – review & editing.
