# Supplementary material for: Effectiveness and safety of vascular intervention plus lenvatinib versus vascular intervention alone for hepatocellular carcinoma patients with portal vein tumor thrombus: a retrospective comparative study
Source: Front Oncol. 2024 Jul 5;14:1431069. doi: 10.3389/fonc.2024.1431069 (PMC11257876; doi:10.3389/fonc.2024.1431069)
Supplement: Supplementary file 1 [file DataSheet_1.docx]

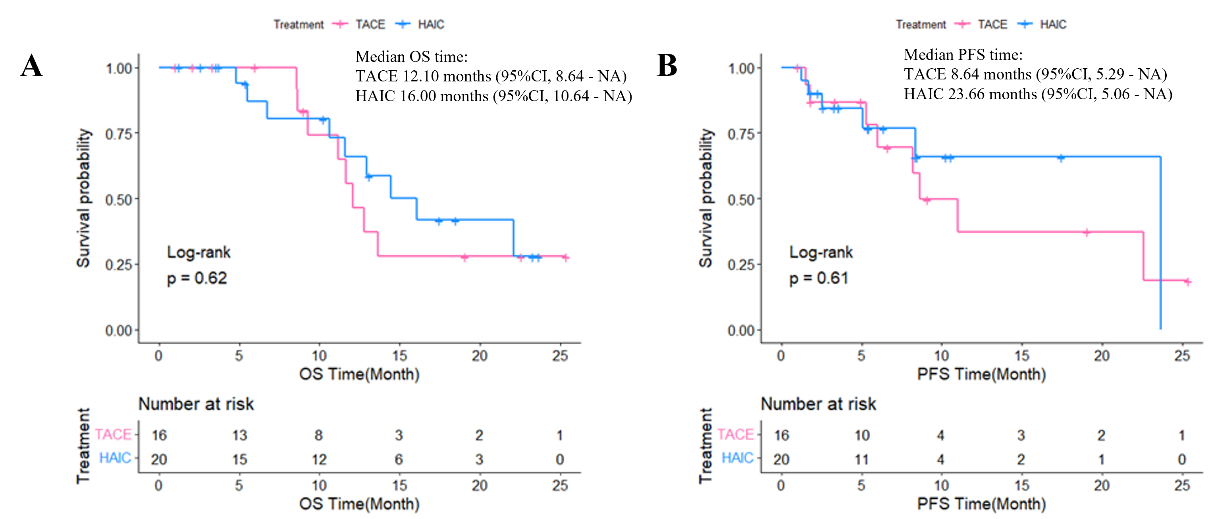


Supplementary Figure 1. Comparison of OS and PFS between patients receiving TACE and HAIC in combination group by Kaplan-Meier method.

Supplementary Table 1. 6-month, 12-month OS rates and PFS rates in two groups

|  | **Group** | **No death / No disease progression** | **Death / Disease progression** | **OS /PFS rates** | ***P*** |
| --- | --- | --- | --- | --- | --- |
| 6months-OS |  |  |  |  | 0.0036 |
|  | Comb | 34 | 2 | 94.44% |  |
|  | Mono | 37 | 19 | 66.07% |  |
| 12months-OS |  |  |  |  | 0.0510 |
|  | Comb | 26 | 10 | 72.22% |  |
|  | Mono | 29 | 27 | 51.79% |  |
| 6months-PFS |  |  |  |  | 0.2083 |
|  | Comb | 28 | 8 | 77.78% |  |
|  | Mono | 36 | 19 | 65.45% |  |
| 12months-PFS |  |  |  |  | 0.6381 |
|  | Comb | 24 | 12 | 66.67% |  |
|  | Mono | 34 | 21 | 61.82% |  |

Comb = Combination Therapy Group; Mono = Monotherapy Group; OS = Overall survival; PFS = Progression-free survival.

Supplementary Table 2. Univariate and multivariate Cox regression analyses for PFS

| **Variable** | **Univariate cox analysis** | | | | **Multivariate cox analysis** | | | |
| --- | --- | --- | --- | --- | --- | --- | --- | --- |
|  | coef | *P* | HR | 95% CI | coef | *P* | HR | 95% CI |
| **Sex**  (F *vs* M) | 0.1655 | 0.7855 | 1.1799 | 0.3583, 3.8852 |  |  |  |  |
| **Age** | -0.0204 | 0.2134 | 0.9798 | 0.9489, 1.0118 |  |  |  |  |
| **BMI** | -0.0664 | 0.1472 | 0.9358 | 0.8554, 1.0237 |  |  |  |  |
| **HBV/HCV infection** |  |  |  |  |  |  |  |  |
| HCV *vs* HBV | -1.0591 | 0.2984 | 0.3468 | 0.0471, 2.5529 |  |  |  |  |
| Absence *vs* HBV | -1.2250 | 0.2406 | 0.2938 | 0.0380, 2.2732 |  |  |  |  |
| **EHS** |  |  |  |  |  |  |  |  |
| LNM *vs* absence | 1.0500 | 0.0116 | 2.8579 | 1.2646, 6.4583 | 1.0541 | 0.0438 | 2.8695 | 1.0298, 7.9956 |
| DM *vs* absence | 0.8143 | 0.0545 | 2.2577 | 0.9845, 5.1776 | 1.2134 | 0.0382 | 3.3651 | 1.0683, 10.5995 |
| **Other treatment before vascular intervention or Lenvatinib** | 0.1390 | 0.7448 | 1.1492 | 0.4976, 2.6539 | -1.1411 | 0.0499 | 0.3195 | 0.1021, 0.9997 |
| **Tumor number** |  |  |  |  |  |  |  |  |
| 2 *vs* 1 | 0.2722 | 0.6630 | 1.3128 | 0.3860, 4.4645 |  |  |  |  |
| >=3 *vs* 1 | 0.1739 | 0.6761 | 1.1899 | 0.5264, 2.6895 |  |  |  |  |
| **Child-Pugh Grade**  (B *vs* A) | 0.0617 | 0.8810 | 1.0637 | 0.4740, 2.3871 |  |  |  |  |
| **PVTT type**  (III/ IV *vs* I/II) | -0.2808 | 0.4947 | 0.7552 | 0.3373, 1.6907 |  |  |  |  |
| **ECOG PS**  (1 *vs* 0) | -0.0487 | 0.8975 | 0.9525 | 0.4544, 1.9966 |  |  |  |  |
| **Max tumor size** | 0.1109 | 0.0080 | 1.1172 | 1.0294, 1.2125 | 0.1473 | 0.0146 | 1.1587 | 1.0295, 1.3042 |
| **Baseline AFP** | 4.46E-06 | 0.0282 | 1 | 1, 1 | 0 | 0.0244 | 1 | 1, 1 |
| **Treatment**  (mono *vs* comb) | 0.7544 | 0.0400 | 2.1264 | 1.0354, 4.3667 | 0.8817 | 0.0899 | 2.4150 | 0.8719, 6.6893 |

AFP = alpha-fetoprotein; BMI = body mass index; Comb = Combination Therapy Group; DM = distant metastases; ECOG = Eastern Cooperative Oncology Group; EHS = extrahepatic spread; HBV = hepatic B virus; HCV = hepatic C virus; IQR = interquartile range; LNM = lymph node metastases; Mono = Monotherapy Group; PS = performance status; PVTT = portal vein tumor thrombus.

Supplementary Table 3. Univariate and multivariate Cox regression analyses for OS

| **Variable** | **Univariate cox analysis** | | | | **Multivariate cox analysis** | | | |
| --- | --- | --- | --- | --- | --- | --- | --- | --- |
|  | coef | *P* | HR | 95% CI | coef | *P* | HR | 95% CI |
| **Sex**  (F *vs* M) | 0.3575 | 0.4105 | 1.4297 | 0.6103, 3.3493 |  |  |  |  |
| **Age** | -0.0162 | 0.1906 | 0.9840 | 0.9605, 1.0081 | 0.0414 | 0.0199 | 1.0423 | 1.0066, 1.0793 |
| **BMI** | -0.0674 | 0.0697 | 0.9348 | 0.8692, 1.0054 |  |  |  |  |
| **HBV/HCV infection** |  |  |  |  |  |  |  |  |
| HCV *vs* HBV | -13.3067 | 0.9961 | 0 | 0, 6.724E+258 |  |  |  |  |
| Absence *vs* HBV | -1.5876 | 0.1162 | 0.2044 | 0.0282, 1.4817 |  |  |  |  |
| **EHS** |  |  |  |  |  |  |  |  |
| LNM *vs* absence | 0.6227 | 0.072 | 1.8639 | 0.9458, 3.6735 |  |  |  |  |
| DM *vs* absence | 0.5788 | 0.0841 | 1.7839 | 0.9251, 3.4400 |  |  |  |  |
| **Other treatment before vascular intervention or Lenvatinib** | 0.3210 | 0.3805 | 1.3785 | 0.6727, 2.8248 |  |  |  |  |
| **Tumor number** |  |  |  |  |  |  |  |  |
| 2 *vs* 1 | 0.8366 | 0.1756 | 2.3086 | 0.6879, 7.7473 | 1.7314 | 0.0170 | 5.6487 | 1.3624, 23.4200 |
| >=3 *vs* 1 | 0.9759 | 0.0122 | 2.6536 | 1.2376, 5.6896 |  |  |  |  |
| **Child-Pugh Grade**  (B *vs* A) | 0.0953 | 0.7712 | 1.0999 | 0.5789, 2.0900 |  |  |  |  |
| **PVTT type**  (III/ IV *vs* I/II) | -0.0900 | 0.7739 | 0.9139 | 0.4945, 1.6890 |  |  |  |  |
| **ECOG PS**  (1 *vs* 0) | -0.4651 | 0.154 | 0.6280 | 0.3313, 1.1906 |  |  |  |  |
| **Max tumor size** | 0.1139 | 0.0001 | 1.1207 | 1.0599, 1.1849 | 0.1328 | 0.0013 | 1.1420 | 1.0536, 1.2380 |
| **Baseline AFP** | 2.62E-06 | 0.0674 | 1.0000 | 1, 1 |  |  |  |  |
| **Treatment**  (mono *vs* comb) | 0.6949 | 0.018 | 2.0034 | 1.1263, 3.5636 | 0.9975 | 0.0114 | 2.7115 | 1.2519, 5.8732 |

AFP = alpha-fetoprotein; BMI = body mass index; Comb = Combination Therapy Group; DM = distant metastases; ECOG = Eastern Cooperative Oncology Group; EHS = extrahepatic spread; HBV = hepatic B virus; HCV = hepatic C virus; IQR = interquartile range; LNM = lymph node metastases; Mono = Monotherapy Group; PS = performance status; PVTT = portal vein tumor thrombus.
